# DISABILITY CHARACTERISTICS AND ABLEIST MICROAGGRESSION EXPERIENCES AMONG AGING ADULTS WITH DISABILITIES

**DOI:** 10.1093/geroni/igad104.2186

**Published:** 2023-12-21

**Authors:** Dylan Serpas, Laura Zettel-Watson, Barbara Cherry, Daniel Ignacio

**Affiliations:** University of South Florida, Tampa, Florida, United States; California State University, Fullerton, Fullerton, California, United States; California State University, Fullerton, Fullerton, California, United States; UCLA BrainSport, Los Angeles, California, United States

## Abstract

People with disabilities (PWD) report experiencing overt and subtle interpersonal disability-based discrimination that is related to poorer functional and health outcomes. Moreover, associations between disability-based discrimination and mental and physical health outcomes are stronger among younger middle-aged than older adults. However, differences in this pattern across disability characteristics and mental health have not been explored. This study investigated associations between disability visibility, age, ableist microaggressions, and mental health symptoms among middle-aged and older adults with disabilities. Participants included 236 middle-aged and older adults recruited online using Amazon’s Mechanical Turk whose ages ranged from 45 to 70 (M=51.44; SD=5.68). Participants were largely non-Hispanic, white, female, employed full-time, graduate educated, and most reported a mobility/physical disability. Participants provided personal demographics and perceived disability visibility (visible, sometimes visible, not visible) and self-reported ableist microaggression experiences and mental health symptoms. A linear multiple regression model controlling for key demographics examined differences in the association between ableist microaggressions and mental health symptoms moderated by age and disability visibility. Results showed a main effect of microaggressions on mental health symptoms (b=.16, p=.044, f2=.02). An interaction effect was found between age and microaggressions (b=.02, p=.003) indicating that as age increased, the association between microaggressions and mental health symptoms increased as disability visibility decreased. Findings illustrate the importance of considering disability characteristics when evaluating subtle disability discrimination among aging adults. Disability discrimination impedes health outcomes and equity, and future research is needed to explore stigma-reduction interventions at local and policy levels.

